# The C-Terminal Sequence of RhoB Directs Protein Degradation through an Endo-Lysosomal Pathway

**DOI:** 10.1371/journal.pone.0008117

**Published:** 2009-12-02

**Authors:** Dolores Pérez-Sala, Patricia Boya, Irene Ramos, Mónica Herrera, Konstantinos Stamatakis

**Affiliations:** 1 Department of Chemical and Physical Biology, Centro de Investigaciones Biológicas, Consejo Superior de Investigaciones Científicas, Madrid, Spain; 2 Department of Molecular and Cellular Medicine, Centro de Investigaciones Biológicas, Consejo Superior de Investigaciones Científicas, Madrid, Spain; University of Pittsburgh, United States of America

## Abstract

**Background:**

Protein degradation is essential for cell homeostasis. Targeting of proteins for degradation is often achieved by specific protein sequences or posttranslational modifications such as ubiquitination.

**Methodology/Principal Findings:**

By using biochemical and genetic tools we have monitored the localization and degradation of endogenous and chimeric proteins in live primary cells by confocal microscopy and ultra-structural analysis. Here we identify an eight amino acid sequence from the C-terminus of the short-lived GTPase RhoB that directs the rapid degradation of both RhoB and chimeric proteins bearing this sequence through a lysosomal pathway. Elucidation of the RhoB degradation pathway unveils a mechanism dependent on protein isoprenylation and palmitoylation that involves sorting of the protein into multivesicular bodies, mediated by the ESCRT machinery. Moreover, RhoB sorting is regulated by late endosome specific lipid dynamics and is altered in human genetic lipid traffic disease.

**Conclusions/Significance:**

Our findings characterize a short-lived cytosolic protein that is degraded through a lysosomal pathway. In addition, we define a novel motif for protein sorting and rapid degradation, which allows controlling protein levels by means of clinically used drugs.

## Introduction

The degradation of cellular components plays a key role in the maintenance of cellular functions. Protein degradation serves regulatory roles in cell cycle and signalling and provides amino acid supply for protein synthesis. The main proteolytic systems in the cell are the proteasome machinery and the lysosomal pathway. Whereas the proteasome has been mainly involved in the rapid degradation of regulatory or misfolded proteins [1], lysosomes are the sites where several degradation pathways converge, including endocytic and autophagic pathways [2].

GTPases of the Rho family play crucial roles in the regulation of the actin cytoskeleton, cell survival and gene expression. Among Rho proteins, RhoB displays unique functions in the control of endocytic traffic, influencing the sorting of signalling kinases [3]–[5] and growth factor receptors [4], [6]. This has important consequences for growth factor signalling, cell survival and proliferation, contributing to the proposed role of RhoB in tumor suppression [7], [8]. RhoB is also unique among Rho proteins due to its short half life, which has been estimated in 2–3 h [9], [10]. RhoB is an immediate early gene which is rapidly induced by DNA damage or growth factors [11], [12]. Thus, maintaining a high RhoB turnover is important to ensure the rapid response of RhoB levels to various stimuli. However, the molecular basis for RhoB instability has not been elucidated.

From a structural point of view, RhoB is highly homologous to RhoA. However, whereas RhoA is retained in the cytosol bound to RhoGDI under resting conditions [13], RhoB is mainly a membrane-associated protein, even in non-stimulated cells [10], and both plasma membrane and endosomal localizations of RhoB have been reported [14], [15]. The structural basis for these differences resides in the hypervariable C-terminal domain of these proteins. Whereas RhoA is geranylgeranylated and possesses polybasic sequence, RhoB presents two palmitoylated cysteine residues (C189 and C192) close to the isoprenylation motif [16]. Hypervariable domains of monomeric GTPases of the Rho and Ras families display unique features including distinct distributions of charged amino acids, phosphorylation sites and lipid moieties [17], [18], which mediate specific membrane localization and orientation, traffic and interaction with effectors [19], [20]. However, the role of hypervariable domains of G proteins in protein stability is poorly understood.

Here we have explored the cellular pathways involved in RhoB degradation and the structural determinants directing this process. Our results reveal several novel striking features of RhoB: 1) this short-lived protein is degraded through a lysosomal pathway; 2) both, isoprenylation and palmitoylation are required for RhoB degradation; and 3) the last eight amino acids of RhoB, which comprise the posttranslational modification motif, are sufficient to direct the endo-lysosomal degradation of chimeric proteins. Taken together, our observations delineate a novel pathway for protein degradation.

## Results

### RhoB Is Degraded through a Lysosomal Pathway

**Figure 1:**
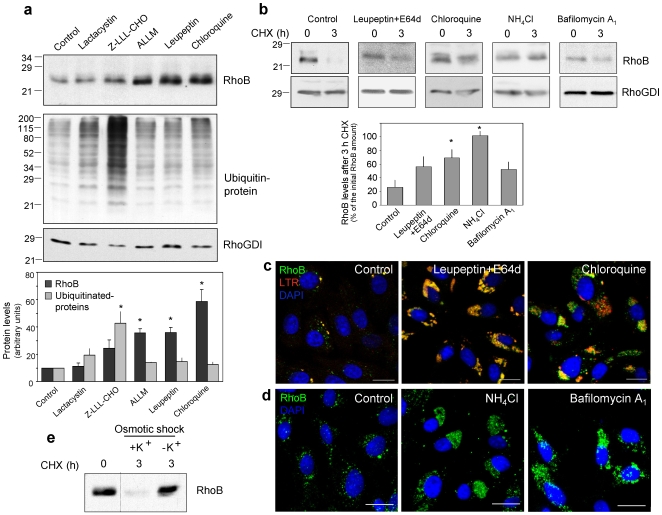
RhoB is degraded through a lysosomal pathway. (**a**) Confluent serum-deprived bovine aortic endothelial cells (BAEC) were treated with 10 µM lactacystin, 50 nM Z-LLL-CHO, 10 µM ALLM, 50 µg/ml leupeptin or 10 µM chloroquine for 24 h. Cell lysates (20 µg of protein) were analyzed by Western blot. RhoB and ubiquitinated protein levels corrected by RhoGDI levels are shown as average values ± SEM of three experiments (*p<0.05 vs control). (**b**) BAEC were pre-treated with the inhibitors for 30 min. NH_4_Cl and bafilomycin A_1_ were added at 30 mM and 5 nM, final concentrations, respectively. RhoB levels were assessed before and after treatment with CHX (20 µg/ml). Levels of RhoGDI were used as control. The percentage of RhoB remaining after 3 h CHX treatment is shown as average values ± SEM of at least three different experiments (*p<0.05 vs control). (**c**) BAEC were treated with 50 µg/ml leupeptin plus 5 µg/ml E64d or 10 µM chloroquine for 24 h and visualized by immunofluorescence (IF) of endogenous RhoB (green) and staining with 500 nM LTR (red) and DAPI (blue). Images shown are single confocal sections. Bar, 20 µm. (**d**) BAEC were treated with 30 mM NH_4_Cl or 5 nM bafilomycin A_1_ for 24 h and fixed. Nuclei were stained with DAPI (blue) and RhoB (green) was detected by IF and confocal microscopy. Images are shown as maximal projections of confocal planes of 0.5 µm Z-sections. Bar, 20 µm. (**e**) BAEC subjected to osmotic shock for 5 min were allowed to recover in the absence or presence of 10 mM potassium chloride (K^+^) for 3 h, during which CHX (20 µg/ml) was present to block new protein synthesis. RhoB levels were assessed by Western blot.

Protein degradation is crucial for cell homeostasis. The main pathways for protein degradation are the ubiquitin proteasome system and the lysosomal pathway. To elucidate the involvement of these pathways in RhoB turnover, we used a battery of protease inhibitors with different specificity towards both pathways (Fig. 1a). The highly selective proteasome inhibitor lactacystin, did not increase RhoB, whereas Z-LLL-CHO, which is less specific [21], moderately increased RhoB levels. ALLM, a calpain-2 inhibitor, and leupeptin, a thiol and serine protease inhibitor clearly increased RhoB. Interestingly, the most effective compound was chloroquine, an inhibitor of lysosomal acidification. Ubiquitinated protein levels, indicative of proteasome inhibition, did not correlate with increases in RhoB. None of the inhibitors increased the levels of RhoGDI, which was used as control (Fig. 1a). Moreover, inhibition of lysosomal degradation stabilized RhoB. RhoB protein levels rapidly decayed in control cells upon inhibition of protein synthesis with cycloheximide (CHX) (Fig. 1b). However, pre-treatment with thiol protease inhibitors, leupeptin plus E64d, or with chloroquine stabilized RhoB, suggesting that RhoB is rapidly degraded by lysosomal proteases. Interestingly, RhoB stabilization was also observed after disrupting the endo-lysosomal pathway through various strategies including reduction of lysosomal acidification by NH_4_Cl or inhibition of the vacuolar H^+^-ATPase with bafilomycin A_1_ (Fig. 1b).

RhoB showed a characteristic punctuate pattern in control cells (Fig. 1c). However, cells treated with inhibitors of lysosomal degradation showed large accumulations of RhoB that were also stained with the fluorescent probe for acidic vesicles Lysotracker Red (LTR). RhoB co-localized with the lysosomal protease cathepsin B, both in control and in chloroquine-treated cells (Fig. S1). NH_4_Cl and bafilomycin A_1_ also induced marked RhoB accumulation (Fig. 1d). Interestingly, osmotic shock followed by potassium depletion, which disrupts both plasma and intracellular membrane-associated clathrin lattices [22] completely stabilized RhoB (Fig. 1e).

The major pathway leading to lysosomal degradation of intracellular components, including long-lived proteins and organelles, is macroautophagy [23]. Blocking autophagy by deletion of the Atg5 gene, as in Atg5(−/−) MEFs or by means of the classical inhibitor 3-methyladenine failed to increase RhoB levels or stability (Fig. S2). Thus, RhoB stabilization by inhibitors of lysosomal proteolysis is not the result of a general disruption of vesicular traffic or of the autophagic pathway, but of selective events in the endo-lysosomal pathway.

**Figure 2:**
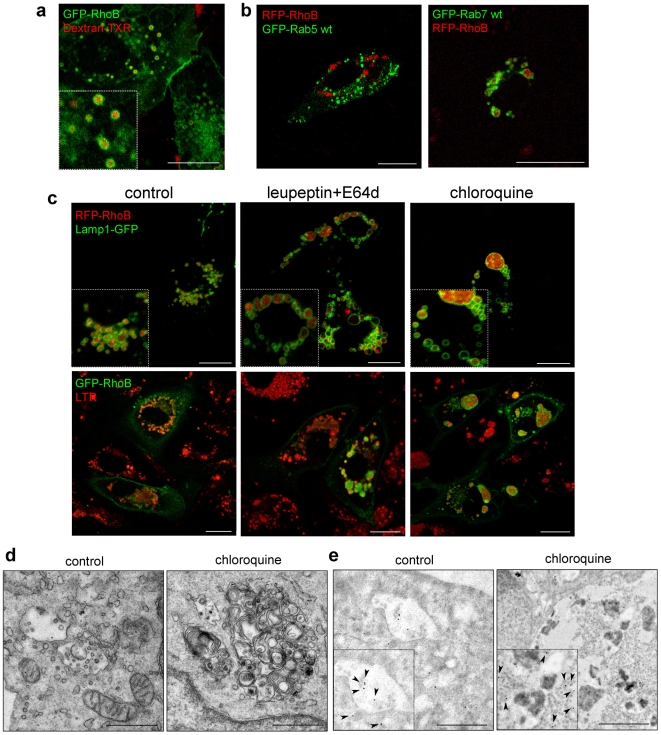
Localization of RhoB along the endo-lysosomal pathway. (**a**) BAEC transiently transfected with GFP-RhoB were incubated with Texas Red-labeled dextran (Dextran-TXR) for 5 min and visualized by confocal microscopy after 1 h. Bar, 20 µm. (**b**) BAEC were transiently transfected with the indicated constructs and 24 h later live cells were observed by confocal microscopy. Bars, 15 µm. (**c**) BAEC were transfected with GFP-RhoB, RFP-RhoB and/or Lamp1-GFP and treated 24 h later with leupeptin plus E64d or chloroquine for further 24 h. When indicated, cells were incubated with 25 nM LTR for 15 min. Live cells were observed by confocal microscopy. Bars, 15 µm. (**d**) Transmission EM of MVB from control and chloroquine treated BAEC. Bars, 1 µm. (**e**) IEM analysis of GFP-RhoB in control and choroquine-treated cells by staining with anti-GFP antibody. Bars, 1 µm.

A higher resolution of RhoB-positive endosomal structures could be achieved by confocal microscopy in live cells. Similarly to endogenous RhoB, GFP-RhoB and RFP-RhoB localized to intracellular vesicles and to the plasma membrane (Fig. 2a,b). The localization of RhoB along the endocytic pathway was confirmed by its presence on vesicles containing endocytosed dextran (Fig. 2a). Interestingly, RhoB was not associated with GFP-Rab5-positive structures but was clearly observed inside vesicles delimited by Rab7, a marker of late endosomes and lysosomes (Fig. 2b). RFP-RhoB was clearly localized inside Lamp1-GFP positive vesicles and GFP-RhoB-positive vesicles showed LTR staining (Fig. 2c). Thus, RhoB is associated with late-endosomes/lysosomes. Interestingly, blocking lysosomal degradation with leupeptin plus E64d or with chloroquine induced the accumulation of RhoB constructs in enlarged lysosomal vesicles (Fig. 2c and S3a). Remarkably, RhoB distribution inside the vesicles showed a patchy appearance, indicative of its presence in intravesicular structures. Indeed, a greater magnification of these dilated compartments, either in GFP-RhoB or Lamp1-GFP transfected cells, revealed multiple intraluminal vesicles, typical of multivesicular bodies (MVB) (Fig. S3b). Electron microscopy (EM) clearly showed the formation of dilated multivesicular structures in chloroquine-treated cells, that could correspond to MVB or to late endosome/lysosome hybrids [24] (Fig. 2d). Immuno-EM (IEM) confirmed the presence of GFP-RhoB in the intraluminal vesicles of MVB both in control and in chloroquine-treated cells (Fig. 2e). Taken together, these results suggest that RhoB, localized in late endosomes/MVB may be delivered to the luminal space and be degraded in this compartment and/or in lysosomes.

### Specific Lipids Regulate RhoB Accumulation into MVB

**Figure 3:**
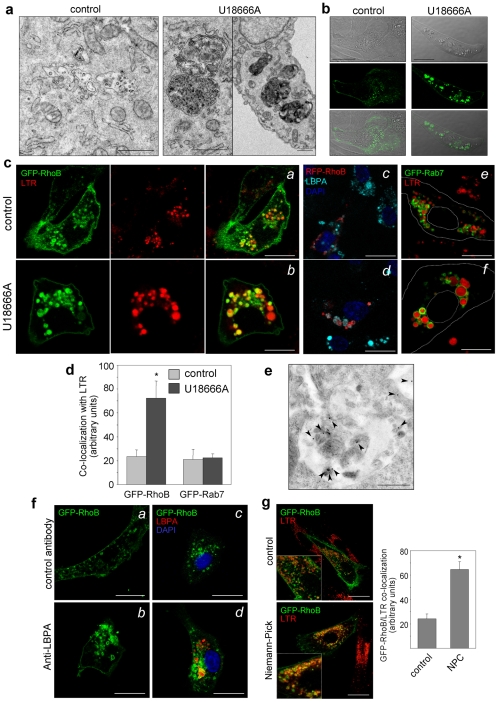
Effect of endosomal lipid dynamics on RhoB distribution. (**a**) BAEC were treated with vehicle or 10 µM U18666A for 24 h, fixed and analyzed by transmission EM. Bars, 1 µm. (**b**) Confocal microscopy GFP-RhoB-transfected cells treated as in A. DIC, fluorescent image and their overlay are shown. Bars, 20 µm. (**c**) BAEC were transfected with the indicated constructs and treated with vehicle or U18666A; (*a,b*) and (*e,f*), confocal microscopy of live cells incubated with 25 nM LTR; cell contours are shown by dotted lines in (*e,f*); (*c,d*) distribution of LBPA observed by IF. Nuclei were stained with DAPI. Bars, 10 µm. (**d**) The degree of internalization of GFP-RhoB or GFP-Rab7 into U18666A-induced structures was estimated from co-localization of the corresponding constructs with LTR and it is shown as average values ± SEM from three independent experiments (*p<0.05 vs control). (**e**) BAEC transfected with GFP-RhoB and treated with U18666A were analyzed by IEM with anti-GFP antibodies. Bar, 0.5 µm. (**f**) Effect of anti-LBPA on RhoB distribution. BAEC transfected with GFP-RhoB were incubated with a control antibody or with anti-LBPA (hybridoma supernatant diluted 1∶1 in culture medium) for 20 h; (*a,b*) live cells observed by confocal microscopy; (*c,d*) accumulation of LBPA observed by IF with anti-mouse Ig antibody. Bars, 20 µm. (**g**) Control or NPC fibroblasts were transfected with GFP-RhoB. Co-localization with LTR was assessed in live cells. Bars, 20 µm. Co-localization is shown as average values ± SEM of determinations from three experiments (*p<0.05 vs control).

The biogenesis of MVB from late endosomes occurs through a complex mechanism involving protein and lipid-mediated processes. Lipid composition and dynamics are crucial for MVB biogenesis [25]. Thus, we explored whether altering late endosome lipids affected RhoB distribution. For this we used the compound U18666A, which causes the accumulation of the late endosome-specific lipid, 2,2′-dioleoyl lysobisphosphatidic acid (LBPA) and cholesterol in late endosomal membranes [26], [27] and promotes their invagination [28]. U18666A induced a massive accumulation of membranes and/or vesicles inside late endosomes giving rise to electron-dense structures (Fig. 3a), that were also visible by differential interference contrast imaging (DIC) (Fig. 3b). Remarkably, U18666A-induced dense MVB showed an intense accumulation of GFP-RhoB (Fig. 3b). RhoB co-localized with LTR and LBPA inside U18666A-induced structures (Fig. 3c *a–d*). In contrast, Rab7, which is bi-geranylgeranylated, was retained at the limiting membranes (Fig. 3c *e,f*). Thus, disruption of late endosome lipid dynamics promotes a selective trapping of RhoB in these structures. The presence of GFP-RhoB in the intraluminal membranes of U18666A-induced dense MVB was confirmed by IEM (Fig. 3e). U18666A did not increase RhoB levels or protein stability (Fig. S4), suggesting that RhoB degradation is not impaired in U18666A-treated cells.

LBPA plays a key role in membrane invagination leading to MVB formation by promoting luminal vesicle formation due to its cone-shaped structure [29] and controls late endosome cholesterol dynamics [26]. The role of LBPA in MVB invagination can be specifically tackled in vivo by treatment with an anti-LBPA antibody, which is endocytosed and accumulates in late endosomes blocking LBPA-dependent dynamics [26]. Anti-LBPA induced a marked distortion of GFP-RhoB-positive endosomes that appeared as dilated structures containing anti-LBPA (Fig. 3f), confirming that late endosome-specific lipids regulate RhoB sorting into MVB.

Genetic alterations of the endo-lysosomal pathway cause various severe human diseases. The dynamics of late endosome-specific lipids is profoundly altered in Niemann-Pick type C disease [26], a neurodegenerative disorder in which a mutation in the NPC1 protein, a regulator of vesicular cholesterol transport [30], causes massive accumulation of cholesterol and LBPA in late endosomes. Using fibroblasts from NPC1 patients, we observed an increase in both, the size of RhoB and LTR positive vesicles and of RhoB-LTR co-localization with respect to fibroblasts from control subjects (Fig. 3g). NPC fibroblasts also showed a marked increase in endosomal cholesterol content, as revealed by filipin staining, which co-localized with RhoB (Fig. S5). These findings show that RhoB distribution is altered in a relevant pathological condition affecting late-endosome lipid dynamics.

### RhoB Sorting into MVB Requires Integrity of the ESCRT Machinery

**Figure 4:**
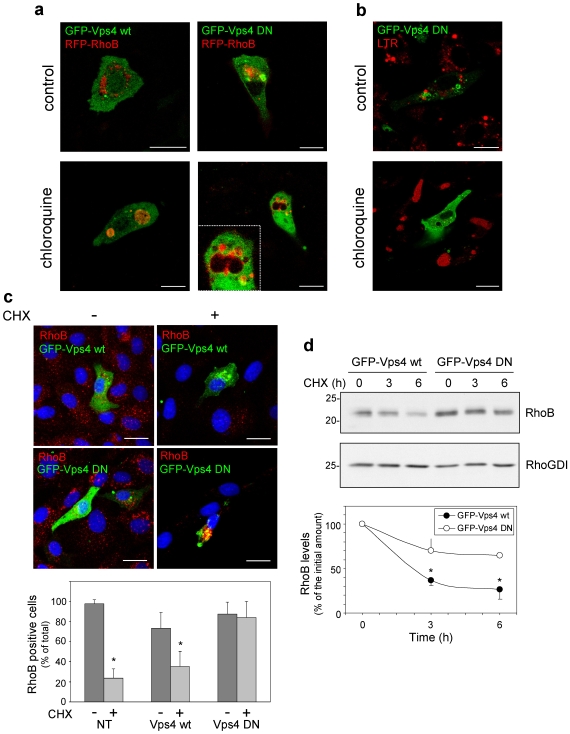
Effect of Vps4 wt and DN on RhoB localization and stability. (**a–b**) BAEC were transfected with GFP-Vps4 wt or DN, as indicated, and 24 h later, treated in the absence or presence of 10 µM chloroquine for 24 h. In (**b**), 25 nM LTR was added 15 min before observation of live cells by confocal microscopy. Bars, 15 µm. (**c**) BAEC were transfected as above and 24 later placed in serum-free medium. After 24 h, control (0 time, -) cells were fixed, and the rest were treated with 20 µg/ml CHX for 8 h (+) to allow degradation of endogenous RhoB, before fixation. Endogenous RhoB (in red) was detected by IF. Nuclei were stained with DAPI. Images shown are overall projections. Bars, 20 µm. The lower panel shows the proportion of RhoB-positive cells among non-transfected (NT) cells or among cells transfected with the indicated constructs before (−) or after CHX (+) treatment. At least 40 cells of each type were monitored from four independent experiments. Results are shown as average values ± SEM (*p<0.05 vs the same condition in the absence of CHX). (**d**) COS7 cells were transfected with GFP-Vps4 wt or DN, as indicated, and 24 h later split in three dishes for treatment with CHX for the indicated times. RhoB and RhoGDI levels were assessed by Western blot. Results shown are average values ± SEM from three experiments (*p<0.05 vs GFP-Vps4 DN at the same time points).

MVB formation involves assembly of the multiprotein complexes known as ESCRT on the late endosomal membrane. Subsequently, recruitment of the ATPase Vps4 and ATP hydrolysis drives the dissociation of the ESCRT machinery, and allows the intraluminal release of vesicles formed by endosomal membrane invagination [31]. Vps4 transiently associates with late endosomes and is mainly cytosolic. In contrast, ATPase-defective Vps4 mutants fail to dissociate from these membranes and severely impair MVB sorting [32]. We observed that whereas GFP-Vps4 wt did not alter RhoB distribution in control or in chloroquine-treated cells (Fig. 4a), the ATPase-defective mutant GFP-Vps4 E223Q, which acts as a dominant negative (DN) [32], drastically reduced the formation of RhoB-containing dilated MVB elicited by chloroquine. Instead, RhoB appeared in small vesicles or retained at the edge of large vesicles (Fig. 4a). Vps4 DN also impaired the formation of chloroquine-elicited enlarged LTR-positive vesicles (Fig. 4b). We next explored the role of Vps4 in RhoB degradation (Fig. 4c,d). First, BAEC were transfected with the constructs of interest and incubated with CHX under conditions resulting in the degradation of most of endogenous RhoB protein. Due to low transfection efficiency, the presence of RhoB was assessed in transfected and non-transfected (NT) cells by IF. After CHX treatment, the proportion of RhoB positive cells was clearly reduced in the cases non-transfected (NT) cells and cells transfected with Vps4 wt but was preserved in cells transfected with Vps4 DN (Fig. 4c). Levels of RhoGDI did not decrease after CHX treatment (Fig. S6). The effect of Vps4 on RhoB levels was determined in COS7 cells, which allow high transfection efficiency. As it is shown in Fig. 4d, the decay in RhoB levels upon CHX treatment was slower in Vps4 DN-transfected than in Vps4 wt-transfected cells. Taken together these results indicate that blocking Vps4 function prevents both RhoB sorting into MVB and degradation.

### The C-Terminal Sequence of RhoB (-CINCCKVL) Acts as a Signal for Protein Endo-Lysosomal Localization and Degradation

**Figure 5:**
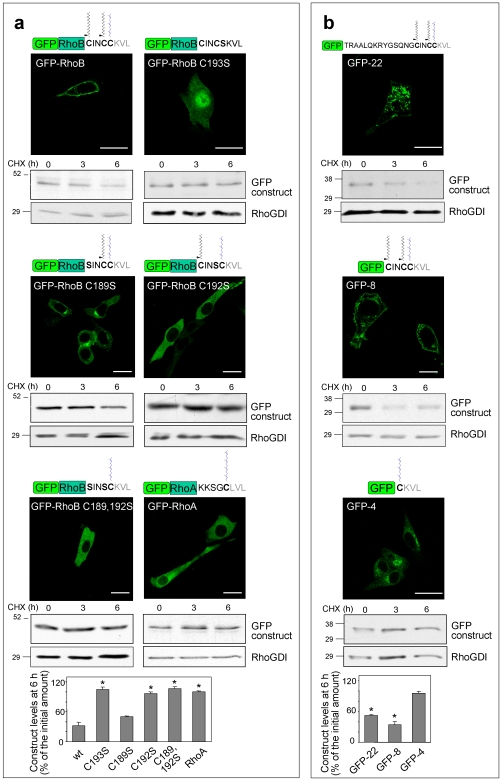
Structural determinants involved in the subcellular localization and degradation of GFP-RhoB. (**a,b**) NIH-3T3 fibroblasts were transiently transfected with the indicated GFP constructs. 24 h after transfection, the distribution of fluorescent constructs was assessed by confocal microscopy of live cells. Bars, 20 µm. The putative posttranslational modifications of each construct are shown schematically above the panels. Construct stability was assessed by blocking protein synthesis with CHX and estimating the remaining levels by Western blot with anti-GFP antibody. Blots show the levels of the constructs of interest and of RhoGDI, as control. Lower panels show the construct levels remaining after 6 h treatment with CHX. wt, GFP-RhoB; C193S, GFP-RhoB C193S; C189S, GFP-RhoB C189S; C192S, GFP-RhoB C192S; C189,192S, GFP-RhoB C189,192S; RhoA, GFP-RhoA. Results are shown as average values ± SEM of three different experiments (*p<0.05 vs wt levels in (**a**) and vs GFP-4 levels in (**b**)).

Lipid modifications of G proteins are key for their specific subcellular localization. To assess the relative importance of the individual lipidic modifications in RhoB localization and stability we generated site-specific mutants. As we reported previously [10], an isoprenylation-deficient mutant, RhoB C193S, failed to associate with cellular membranes and its turnover was completely inhibited (Fig. 5a). Mutation of the first palmitoylation site (C189S), induced a diffuse cytosolic distribution with a perinuclear accumulation and partially impaired degradation, whereas mutation of the second palmitoylation site (C192S) completely stabilized RhoB, same as the double C189,192S mutation. All palmitoylation mutants excluded the nucleus, indicating that isoprenylation is preserved. Thus, both palmitoylation sites contribute to the localization and degradation of RhoB, although to different extents. We next seek to modulate RhoB turnover pharmacologically by interfering with isoprenylation and palmitoylation. Both simvastatin, an isoprenoid biosynthesis inhibitor, and 2-bromopalmitate, a palmitoylation inhibitor, increased the levels of GFP-RhoB and endogenous RhoB, but not of RhoA (Fig. S7). Moreover, short-term treatment (3 h) with either inhibitor elicited the appearance of diffusely distributed GFP-RhoB, which excluded the nucleus in the case of 2-BP, but not in the case of simvastatin-treated cells (Fig. S7c). Since isoprenylation is irreversible, the effect of simvastatin is consistent with the diffuse localization of the newly synthesized non-processed protein. Thus, isoprenylation and palmitoylation may act as switches for RhoB degradation.

RhoA is 86% identical to RhoB differing mainly in the C-terminal sequence. As shown in Fig. 5a, GFP-RhoA was cytosolic and stable, suggesting that RhoB C-terminus contains the structural determinants for degradation. To test this hypothesis, we prepared chimeric proteins of GFP with several short sequences from RhoB C-terminus (Fig. 5b). Interestingly, chimeric proteins containing only the last 22, or even the last 8 amino acids of RhoB, showed a GFP-RhoB-like distribution, and were not stable. In contrast, the fusion protein containing only the CAAX box appeared diffuse with a perinuclear accumulation, and was not detectably degraded. Thus, the isoprenylation motif alone is not sufficient, but a sequence containing the isoprenylation and the two palmitoylation sites of RhoB (CINCCKVL) efficiently directs protein localization and degradation.

**Figure 6:**
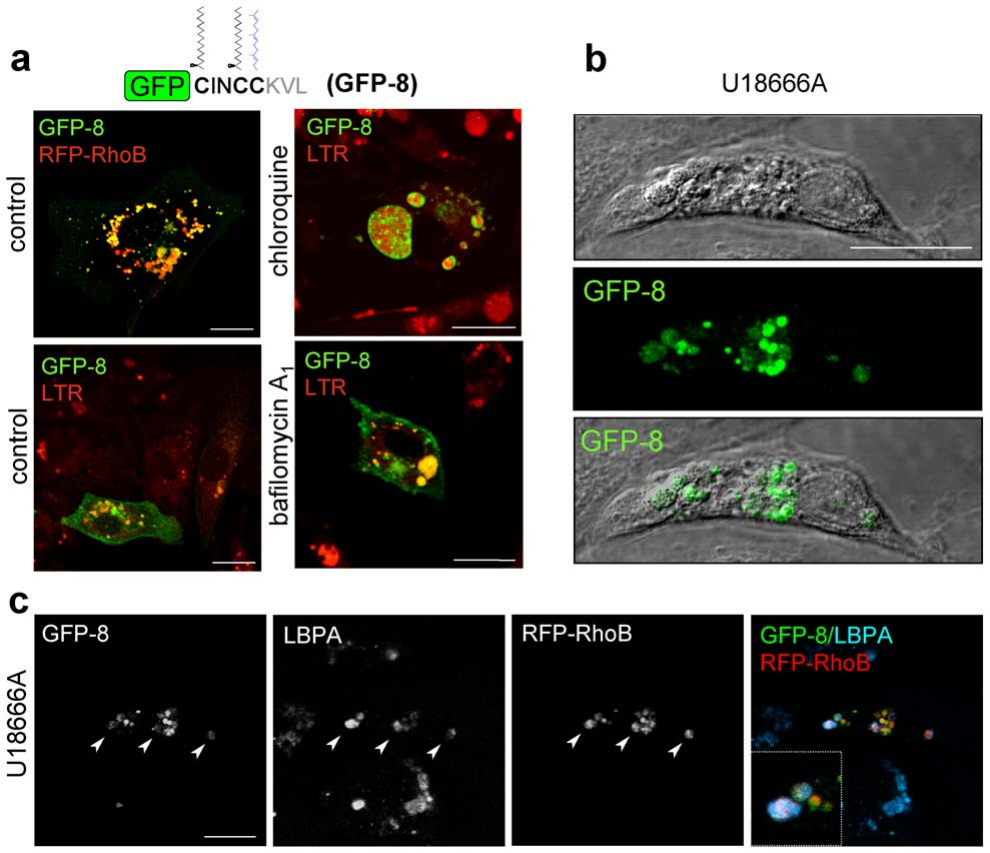
Localization of GFP-CINCCKVL in endo-lysosomal compartments. (**a**) BAEC transfected with RFP-RhoB and/or GFP-CINCCKVL (GFP-8) were treated with vehicle, 10 µM chloroquine or 5 nM bafilomycin A_1_, incubated with 25 nM LTR when indicated and visualized live by confocal microscopy. (**b**) BAEC transfected with GFP-8 were treated with 10 µM U18666A for 24 h. DIC and fluorescence images showing dense lysosomes and GFP-8 accumulations are shown. (**c**) BAEC transfected with GFP-8 and RFP-RhoB and treated with U18666A as above were fixed and co-localization of GFP-8, RFP-RhoB and LBPA was detected by IF. Bars, 20 µm.

In order to investigate if this sequence was targeting chimeric proteins for degradation through a lysosomal pathway, we assessed the localization of the GFP-CINCCKVL construct, termed GFP-8, in the presence of lysosomal inhibitors. GFP-8 significantly co-localized with RFP-RhoB and LTR in control cells (Fig. 6a). Moreover, in cells treated with chloroquine or with bafilomycin A_1_ GFP-8 was retained inside LTR-positive dilated vesicles, likewise GFP-RhoB/LTR (Fig. 2c). Furthermore, GFP-8 accumulated in U18666A-induced dense MVB visible by DIC (Fig. 6b), where it co-localized with RFP-RhoB and LBPA (Fig. 6c). Thus, GFP-8 is internalized into MVB by mechanisms similar to those operating on RhoB.

### Targeting Stable Proteins for Rapid Lysosomal Degradation through the CINCCKVL Sequence

**Figure 7:**
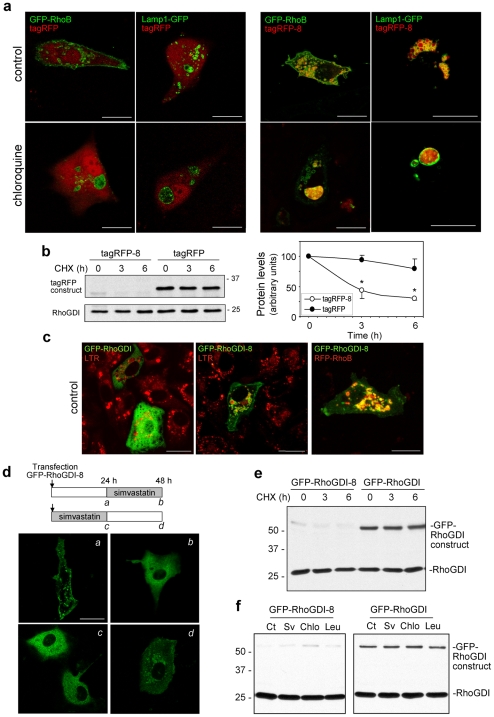
Subcellular localization and stability of chimeric proteins bearing -CINCCKVL C-terminal sequences. (**a**) BAEC were co-transfected with tagRFP or tagRFP-CINCCKVL (tagRFP-8) and GFP-RhoB or Lamp1-GFP, as indicated, treated for 24 h in the absence or presence of 10 µM chloroquine and directly visualized by confocal microscopy. Images shown are individual confocal sections. Bars, 20 µm. (**b**) The stability of tagRFP and tagRFP-8 was assessed in BAEC as described in Fig. 5, using an anti-tagRFP antibody. Results are shown as average values ± SD of at least three determinations (*p<0.05 vs tagRFP levels at the same time points). (**c**) BAEC were transfected with GFP-RhoGDI, GFP-RhoGDI-8 and/or RFP-RhoB, incubated with 25 nM LTR, as indicated, and directly observed by confocal microscopy. Bar, 20 µm. (**d**) BAEC were transfected with GFP-RhoGDI-8 in the absence (*a*) or presence (*c*) of 10 µM simvastatin. 24 h after transfection, confocal images of live cells were obtained (*a*, *c*). At that point, simvastatin was withdrawn from (*c*) and added to (*a*). After a 24 h additional incubation images of live cells were taken (*b*, *d*). Bar, 20 µm. (**e,f**) BAEC were transiently transfected with GFP-RhoGDI-8 or GFP-RhoGDI. In (**e**) protein synthesis was blocked by CHX addition. In (**f**) BAEC were treated with the indicated agents for 24 h. Levels of GFP-RhoGDI-8, GFP-RhoGDI and endogenous RhoGDI, were assessed by Western blot. Sv, simvastatin; Chlo, chloroquine; Leu, leupeptin.

To assess the ability of the CINCCKVL sequence to induce endo-lysosomal localization and rapid degradation of stable cytosolic proteins we created additional chimeras. TagRFP, a monomeric protein only 29% identical to GFP, was diffuse and did not co-localize with GFP-RhoB or Lamp1-GFP (Fig. 7a). In contrast, the chimeric protein tagRFP-CINCCKVL (tagRFP-8) co-localized with GFP-RhoB-positive vesicles and appeared inside Lamp1-GFP-delimited vesicles. In chloroquine-treated cells, tagRFP remained excluded from RhoB-containing and Lamp1-delimited dilated MVB, whereas tagRFP-8 was totally accumulated inside. Moreover, whereas tagRFP was stable, tagRFP-8 reached lower levels and was rapidly degraded (estimated half-life of tagRFP-8 ≤3 h; Fig. 7b). As expected, tagRFP-8 migrated faster than tagRFP in SDS-PAGE gels, a feature typical of other isoprenylated and palmitoylated proteins, like RhoB or H-Ras [10], [33].

As shown above, the levels of the cytosolic protein RhoGDI are not altered by agents modulating lysosomal protein degradation or endosomal traffic and they remain stable after inhibition of protein synthesis (Fig. 1). Therefore, we considered it a good candidate to confirm the effect of the addition of the CINCCKVL sequence to its C-terminal end. Whereas GFP-RhoGDI was diffuse, GFP-RhoGDI-CINCCKVL (GFP-RhoGDI-8) was associated with LTR-positive cytosolic vesicles and strongly co-localized with RFP-RhoB (Fig. 7c). Moreover, in chloroquine-treated cells, GFP-RhoGDI was excluded from, whereas GFP-RhoGDI-8 was accumulated in LTR and RhoB-positive dilated vesicles (Fig. S8), thus corroborating the lysosomal localization of the chimeric protein. GFP-RhoGDI-8 lysosomal targeting was sensitive to modulation of isoprenylation. Treatment of cells expressing membrane and vesicle-associated GFP-RhoGDI-8 with simvastatin led to its diffuse localization throughout the cytosol (Fig. 7d, a and *b*). Conversely, when cells were transfected with GFP-RhoGDI-8 in the presence of simvastatin, the protein was completely cytosolic (Fig. 7d *c*) but removal of the statin allowed its appearance in cytosolic vesicles (Fig. 7d *d*).

As observed with other -CINCCKVL chimeric proteins, the levels and the half-life of GFP-RhoGDI-8 were consistently lower than those of GFP-RhoGDI and endogenous RhoGDI (Fig. 7e). Interestingly, simvastatin increased the levels of GFP-RhoGDI-8 but not of GFP-RhoGDI (Fig. 7f). These results indicate that C-terminal processing can act as a switch for the localization and degradation not only of RhoB but of chimeric proteins bearing the CINCCKVL motif. The specific subcellular localization and reduced protein stability of GFP-RhoGDI-8 (half-life ≤3 h) were also observed in stably transfected NIH-3T3 fibroblasts (Fig. S8), ruling out a potential interference of lipid-mediated transfection in the effects observed. Finally, we confirmed that the CINCCKVL motif rendered GFP-RhoGDI-8 susceptible to modulation by lysosomal proteolysis inhibitors. Leupeptin and chloroquine increased GFP-RhoGDI-8 levels, whereas GFP-RhoGDI and endogenous RhoGDI remained unchanged (Fig. 7f). Taken together, these results show that the CINCCKVL sequence is capable of promoting a RhoB-like localization and degradation rate when fused to the C-terminal end of unrelated proteins like GFP, tagRFP and GFP-RhoGDI. Moreover, both the levels and distribution of the chimeric proteins can be regulated pharmacologically by means of widely used drugs, such as statins.

## Discussion

Rapid protein degradation contributes to the tight control of RhoB levels. The study of the mechanisms responsible for RhoB degradation points to the existence of a degradation pathway (schematized in Fig. S9) by which RhoB, a cytosolic protein, is delivered into MVB and is degraded in acidic compartments. This process depends on the function of proteins of the Vps machinery and on RhoB posttranslational modification, which mediates its association with late endosomes. Moreover, a sequence of only eight amino acids comprising the RhoB lipidation motif can “pull” other proteins towards an endo-lysosomal compartment for rapid degradation.

The finding that the lysosomal pathway achieves the rapid degradation of RhoB is relevant because the proteasome is considered responsible for the degradation of most short-lived proteins [1]. Autophagy constitutes the major pathway leading to lysosomal degradation [23]. Our results indicate that macroautophagy is not responsible for RhoB degradation. This is consistent with observations indicating that autophagy does not play an obvious part in the degradation of short-lived proteins, even under serum deprivation conditions [34]. On the other hand, the CINCCKVL sequence does not respond to the any of the consensus sequences that elicit chaperone-mediated autophagy [35]. In contrast, our results imply that RhoB reaches lysosomes through the endocytic pathway. RhoB is found in MVB and is strongly accumulated in this compartment when the MVB lipids are selectively altered either by pharmacological means, such as U18666A and endocytosed anti-LPBA antibodies, or by genetic disease. Although these conditions clearly affect MVB membrane dynamics, it is not clear how they would affect lysosomal degradation. Endocytosed anti-LBPA antibodies do not impair EGFR degradation [36], whereas U18666A reduces late endosome acidification. Thus the lack of a detectable effect of this compound on RhoB degradation could be the net result of increased invagination and compromised proteolytic activity [37]. It is interesting to note that lipid-mediated “trapping” of RhoB into MVB is selective since Rab7, which is also associated with the late endosome limiting membrane, is not accumulated in this compartment. This could arise from the strong binding of RhoB to defined endosomal membrane domains resulting in its remaining associated with invaginating membranes. In fact, RhoB extraction from membranes requires high detergent concentrations (unpublished observations).

Given the clear role of MVB membrane invagination in RhoB sorting we used a powerful strategy to block this process. DN Vps4 brings about an overall impairment of the ESCRT machinery and blocks intraluminal vesicle formation [38]. The inhibition of RhoB degradation specifically observed in Vps4 DN-transfected cells confirms that RhoB is degraded through an endo-lysosomal pathway requiring MVB biogenesis.

The observation that palmitoylation appears to play a positive role in RhoB degradation constitutes a singular case since, in mammalian cells, palmitoylation most often targets proteins away from lysosomes, resulting in increased stability [39]–[42]. The CINCCKVL sequence is unique to RhoB as determined by BLAST and is conserved in all species in which the RhoB sequence is available. However, several proteins belonging to the Ras superfamily, including H-Ras, TC10 and Rap2 possess palmitoylated cysteines close to the isoprenylated C-terminus. Nevertheless, these proteins contain specific features in their hypervariable domains including distinct distributions of acidic and basic amino acids and/or spacing of lipidated residues that result in a different subcellular localization in spite of possessing a lipidation motif similar to that of RhoB [20]. More distantly, in yeast, palmitoylation of Vac8 and Ykt6, the latter being also isoprenylated, has been found to direct localization at the vacuole [43], [44], although at present, there is no information on its role on protein stability. Future studies will address whether similar mechanisms may operate for other proteins.

Our results show that the sequence containing RhoB isoprenylation and palmitoylation sites (CINCCKVL) is sufficient to promote a RhoB-like localization and degradation rate when fused to the C-terminal end of several unrelated proteins. Therefore, an interesting aspect of our studies is the potential use of the CINCCKVL sequence to control the localization and cellular levels of proteins of interest. For instance, chimeric proteins bearing this sequence could be potentially used for delivering bioactive components to the endo-lysosomal compartment to modulate lysosomal function, which has broad implications for cell viability, both in carcinogenesis and degenerative processes. Moreover, levels of CICNCKVL-ending proteins can be increased by lysosomal protease inhibitors. This raises the possibility of using these chimeric proteins as reporters of lysosomal proteolytic activity. Importantly, isoprenylation and palmitoylation can act as switches for the lysosomal targeting and degradation of chimeric proteins bearing CINCCKVL C-terminal sequences. Thus, levels of CINCCKVL-ending chimeric proteins can be regulated by commonly used drugs such as simvastatin. Also, given the increasing interest for the clinical use of isoprenylation inhibitors, the potential for use of CINCCKVL contructs for monitoring isoprenylation efficiency in living cells can be hypothesized. Finally, identification of this novel protein degradation sequence may provide valuable tools to study the mechanisms of MVB formation and lysosomal proteolysis in health and disease.

## Materials and Methods

### Cell Culture and Treatments

BAEC were obtained and cultured as previously described [10]. NIH-3T3 fibroblasts or COS7 cells, cultured as described [10] were treated with the indicated agents or vehicle in the absence of serum. Human primary fibroblasts from apparently healthy subjects and Niemann-Pick disease patients were from Coriell Institute for Medical Research (Candem, NJ). MEFs from wt and Atg5(−/−) mice were kindly provided by Dr. N. Mizushima.

### Antibodies and Reagents

Antibodies for Western blot were anti-RhoB (sc-180), anti-RhoGDI (sc-360), anti-RhoA (sc-418), and anti-ubiquitin (sc-8017), from Santa Cruz Biotechnology (Heildelberg, Germany), anti-GFP from Clontech (Palo Alto, CA) and anti-tagRFP from Evrogen (Moscow, Russia). Antibodies for IF were anti-RhoB (sc-8048) and anti-RhoGDI (sc-360), from Santa Cruz Biotechnology, and anti-cathepsin B from Calbiochem. Anti-LBPA was from Echelon Biosciences Inc. (Salt Lake City, UT). Bafilomycin A_1_ was from A.G. Scientific, Inc. (San Diego, CA). LTR was from Molecular Probes (Invitrogen). Simvastatin was a gift form Merck, Sharp & Dohme (Madrid, Spain).

### Plasmids and Transfections

pEGFP-C1 and pDsRed1-C1 were from Clontech. pTagRFP-C was from Evrogen. Human RhoGDI cDNA was from Origene (Rockville, MD). The pcDNA3-HA-RhoB plasmid was generously provided by Dr. G.C. Prendergast. Lamp1-GFP was from Prof. Jennifer Lippincott-Schwartz (NIH, Bethesda, MD). GFP-Rab7 wt was kindly provided by Prof. Cecilia Bucci (University of Copenhagen, Denmark). GFP-Vps4 wt and E334Q were the generous gift of Dr. Philip Woodman (University of Manchester, UK).

The generation of pEGFP-C1-HA-RhoB wt and C193S mutant has been described [10]. Palmitoylation-deficient mutants of RhoB were generated by PCR using oligonucleotides: 5′-GCTCTAGATCATAGCACCTTGCACGAGTTGATGC-3′, 5′-GCTCTAGATCATAGCACCTTGCAGCAGTTGATCGAGCCATTCTG-3′, and 5′-GCTCTAGATCATAGCACCTTGCACGAGTTGATCGAGCCATTCTG-3′ for the C192S, C189S, and C189,192S mutants, respectively. Mutation sites are underlined. Constructs encoding chimeric proteins comprising GFP and various sequences from the RhoB or TC-10 C-terminus, were generated by PCR using pEGFP-C1 as a template. The forward primer was common for all constructs: 5′-GAGTAGAAGCTTATGGTGAGCAAGGGCGAGGAG-3′. Reverse primers were 5′-CCGAATTCTAGAGGACCTTGCACTTGTACAGCTCGTCC-3′, 5′-CCGAATTCTAGAGGACCTTGCAACAGTTGATACACTTGTACAGCTCGTCCATGC-3′ and 5′-CCGAATTCTCACGTAATTAAACAACAGTTGATACACTTGTA CAGCTCGTCCATGC-3′ for generating the GFP-CKVL, GFP-CINCCKVL (GFP-8) and GFP-CINCCLIT (GFP-8T) constructs, respectively. PCR products were digested with HindIII and EcoRI and cloned into pcDNA3.0. The construct encoding GFP plus the last 22 amino acids of RhoB was generated by amplifying GFP with a BssHII site at its 3′ end, using pEGFP-C1 as a template and primers, forward 5′-GAGTAGAAGCTTATGGTGAGCAAGGGCGAGGAG-3′, and reverse 5′-CATCTTGCGCGCGTCTTGTACAGCTCGTCC-3′. The PCR product was subcloned into the HindIII-BssHII sites of pcDNA-RhoB. The BssHII site is located at position 806 of the sequence of the rat RhoB gene (NM_022542). Therefore, this construct contains the C-terminal sequence of RhoB from T175. pRFP-RhoB was generated by cloning HA-RhoB into the BglII-EcoRI sites of pRFP. For generation of the construct encoding tagRFP-CINCCKVL, tagRFP was amplified by PCR using pTagRFP-C as template and oligonucleotides, forward 5′- CCGCTAGCGCTACCGGTCGCCACCATGG-3′ and reverse, 5′- CCGGATCCCTAGAGGACCTTGCAACAGTTGATACAATTAA GTTTGTGCCCCAGTTTGC-3′. The PCR product was subcloned into the NheI-BamHI sites of the pTagRFP-C plasmid. The cDNA of human RhoGDI (Origene Technologies, Inc. Rockville, MD) was amplified with oligonucleotides introducing restriction sites for SacI or Eco RI: forward primer 5′- CCGAGCTCAAATGGCTGAGCAGGAGCCC-3′, reverse primer 5′- CCGAATTCTCAGTCCTTCCAGTCCTTCTTGATGG-3′. The PCR product was cloned into the SacI-EcoRI sites of the pEGFP-C1 plasmid. For generation of GFP-RhoGDI-CINCCKVL, the same strategy was used except that the oligonucleotide 5′- CCGAATTCCTAGAGGACCTTGCAACAGTTGATACAGTCCTTCCAGTCCTTCTTGATGG-3′ was used as reverse primer.

Confluent BAEC or NIH-3T3 fibroblasts or COS7 cells at 80% confluence were transfected with Lipofectamine 2000 (Invitrogen) using 1 µg of construct following the manufacturer instructions.

### Western Blots

SDS-PAGE and Western blot analysis were performed as described [10] using ECL detection (Amersham, Buckinghamshire, UK). Levels of the proteins of interest were estimated by image scanning of non-saturated ECL exposures and corrected by the band intensities of the Coomassie staining of membranes or by the signal given by an antibody against a non-related protein (RhoGDI for most experiments).

### Fluorescence Microscopy

Cells were cultured on glass bottom dishes (Mattek Corp., Ashland, MA), transfected with fluorescent constructs, treated with the indicated agents and visualized directly on a confocal microscope (LEICA DMRE2, Heidelberg, Germany). For IF cells were fixed with 4% paraformaldehyde, permeabilized with 0.1% SDS, and incubated with primary antibodies and secondary Alexa-Fluor conjugated antibodies (Invitrogen), counterstained with DAPI (Invitrogen) and mounted with Fluoromount-G (Southern Biotechnology Associates, Birmingham, AL). Unless otherwise stated, images shown are single Z-section images for co-localization assessment. All experiments were repeated at least three times and representative results are shown.

### Electron Microscopy

EM analysis was carried out at the EM Facility of Centro de Biología Molecular Severo Ochoa (CSIC, Madrid). For conventional EM analysis cell monolayers were fixed in situ with 2% glutaraldehyde in Sörensen phosphate Na/K buffer (pH 7.4). Fixed cells were processed for embedding in Epoxy, TAAB 812 Resin (TAAB Laboratories, Berkshire, England). Subsequently, cells were subjected to postfixation with 1% osmium tetroxide/0.8% potassium ferrocyanide, incubation with 0.15% tanic acid, incubation with 2% uranyl acetate and dehydration in increasing concentrations of ethanol. Infiltration of the resin was accomplished by increasing concentrations of Epon/ethanol. After polymerization of infiltrated samples, ultrathin sections were stained with saturated uranyl acetate and lead citrate and examined at 80 kV in a Jeol JEM-1010 (Tokyo, Japan) electron microscope. For immuno-electron microscopy cells were processed by freeze substitution. Cells were fixed in situ with 4% paraformaldehyde/0.1% glutaraldehyde in 0.1 M phosphate buffer (pH 7.2). Cells were embedded in 10% gelatine, cryoprotected with 30% glycerol and frozen in liquid nitrogen. Freeze substitution was carried out at −85°C in methanol containing 0.5% uranyl acetate in an Automatic Freeze-Substitution system (AFS, Leica). Samples were later infiltrated with Lowicryl HM20 and polymerized by UV irradiation at −45°C for two days. Ultra thin Lowicryl sections were obtained in a Reichert-Jung Ultracut E ultramicrotome. Samples were examined as above. For immunogold labelling the ultra thin sections were placed sequentially on top of drops of the different solutions: TBS (30 mM Tris-HCl, pH 8.0, 150 mM NaCl), 0.2 M ammonium chloride in PBS, blocking buffer (TBS +0.1% BSA and 1% gelatine) and rabbit anti-GFP antibody (Invitrogen) diluted 1∶100 in blocking buffer. After washing, grids were incubated with protein A-15 nm gold conjugate (Cell Microscopy Center. University Medical Center. Utrecht, The Netherlands). After washing, grids were air dried and counterstained with 2% aqueous uranyl acetate and 1% lead citrate. Grids were examined as above.

### Estimation of Protein Stability

Protein half-life was evaluated by time-course experiments in the presence of CHX and Western blot analysis. Stability of the various constructs used was assessed by cell transfection followed by tripsinization to split cells in identical aliquots. After attachment to substrate, cells were treated with CHX and construct levels were estimated by Western blot.

### Quantification and Statistics

The degree of colocalization of the different constructs was analyzed with software from Leica and Scion Corporation for image analysis. Results are presented as average values ± SD or SEM, as indicated. Average values were compared by Student's t-test for unpaired observations.

## Supporting Information

Figure S1 Co-localization of RhoB and lysosomal proteases. (a) The distribution of RhoB (red) and cathepsin B (CB, green) in BAEC treated with vehicle or 10 µM chloroquine for 24 h was assessed by IF, nuclei were stained with DAPI (blue); bar, 20 µm. Graphs represent the fluorescence intensity profile along the lines depicted in the images, showing the points of co-localization. Results are representative of three assays. (b) The percentage of cells showing five or more points of co-localization of RhoB and cathepsin B was calculated by monitoring 40 cells per experimental condition from three different experiments and it is shown as average values ± standard error of mean (SEM).
(6.04 MB TIF)

Figure S2 Effect of autophagy modulation on RhoB levels and degradation. (a) MEFs from wt and Atg5(−/−) mice were incubated with CHX to block new protein synthesis and the levels of endogenous RhoB were assessed at the indicated times by Western blot. (b) BAEC were treated in the absence or presence of 10 mM 3-methyladenine (3-MA) for 24 h and RhoB levels were estimated as above. Dotted lines show sites where lanes from the same gel have been cropped. Results are average values ± SEM of three experiments. (c) BAEC were pretreated with 3-MA for 30 min, after which, CHX was added and levels of RhoB were analyzed at the indicated times. Results are average values ± SEM of three experiments.
(2.29 MB TIF)

Figure S3 Effect of lysosomal inhibitors on the size and pattern of RhoB-positive vesicles. (a) BAEC were transfected with GFP-RhoB as in Fig. 2c and the size distribution of RhoB-positive vesicles was determined by measuring 60 randomly taken RhoB-positive vesicles per experimental condition from three experiments. The proportion of vesicles of the indicated sizes is expressed as percentage of the total. (b) Multivesicular pattern of chloroquine-elicited GFP-RhoB and Lamp1-GFP vesicles in BAEC transfected as in Fig. 2c. Bars, 5 µm.
(1.92 MB TIF)

Figure S4 Effect of U18666A on RhoB stability. BAEC were treated with 10 µM U18666A for 24 h. Levels of endogenous RhoB and of RhoGDI (as control) were assessed by Western blot at the indicated times after CHX addition. Results shown are average values ± SEM of four experiments.
(1.76 MB TIF)

Figure S5 Co-localization of GFP-RhoB with cholesterol in control or NPC fibroblasts. Control or NPC fibroblasts were transfected with GFP-RhoB. Co-localization with cholesterol was evaluated in fixed cells stained with filipin. Bars, 20 µm. Co-localization is shown as average values ± SEM of determinations from three experiments (*p<0.05 vs control).
(3.21 MB TIF)

Figure S6 Levels of RhoGDI in BAEC detected by IF before and after CHX treatment. BAEC were treated as in Fig. 4, and fixed. Endogenous RhoGDI (in red) was detected by IF. Nuclei were stained with DAPI. Images shown are overall projections. Bars, 20 µm.
(4.04 MB TIF)

Figure S7 Effect of inhibitors of isoprenylation and palmitoylation on the levels of endogenous RhoB protein or of RhoB constructs in different cell types. (a) NIH-3T3 fibroblasts were transiently transfected with GFP or with GFP-RhoB, and construct levels were assessed by Western blot with an anti-GFP antibody after 24 h treatment with simvastatin (Sv) or 2-bromopalmitate (2-BP). (b) NIH-3T3 fibroblasts or BAEC were treated as in (a) and the levels of the indicated proteins were assessed by Western blot. Dotted lines show sites where lanes from the same gel have been cropped. (c) BAEC were transiently transfected with GFP-RhoB, treated with simvastatin or 2-BP for 3 h and visualized by live confocal microscopy. Note that in simvastatin-treated cells, GFP-RhoB can be detected in the nucleus, which is indicative of the presence of non isoprenylated construct. In contrast, in 2-BP-treated cells the appearance of GFP-RhoB is more diffuse than in control cells but the nucleus is excluded, as expected from an isoprenylated construct. Bars, 20 µm.
(3.78 MB TIF)

Figure S8 Cellular distribution and stability of GFP-RhoGDI and GFP-RhoGDI-8. (a) BAEC were transiently transfected with the indicated constructs as in Figure 6, treated with 10 µM chloroquine for 24 h and incubated with 25 nM LTR for 15 min before observation of live cells by confocal microscopy. Bar, 20 µm. (b,c) NIH-3T3 fibroblasts were stably transfected with the indicated constructs. Protein stability (b) and subcellular localization of constructs in control and chloroquine-treated cells (c) were assessed as described in Fig. 6, after 30 days in culture. Bar, 20 µm.
(6.51 MB TIF)

Figure S9 Hypothetical model for the degradation pathway of RhoB and -CINCCKVL chimeric proteins. Isoprenylated and palmitoylated RhoB is targeted to intracellular vesicles, mainly Rab7-, Lamp1-positive late endosomes, and to the plasma membrane. The association of the ESCRT complexes on the membrane of late endosomes promotes the invagination of the membrane. The ATPase Vps4 promotes the dissociation of the ESCRT proteins which is required for the intraluminal release of the invaginated vesicles. The late endosome-specific lipid LBPA promotes luminal vesicle formation due to its cone-shaped structure and controls endosome cholesterol content. Our results support a hypothesis according to which late-endosome associated RhoB may be delivered to intraluminal vesicles of MVB in a Vps4- and LBPA-dependent fashion, for degradation in acidic compartments. Inhibition of vesicular acidification with chloroquine or of lysosomal proteases with protease inhibitors results in the formation of dilated MVB in which RhoB accumulates. Genetic or pharmacological disruption of late endosome specific lipid dynamics also alters RhoB sorting. The proposed points of regulation of RhoB targeting to the endo-lysosomal pathway are shown in blue boxes. Based on our experimental observations, -CINCCKVL chimeric proteins would follow a similar pathway for rapid protein degradation. Ub, ubiquitin; GFR, growth factor receptor; GF, growth factor.
(4.56 MB TIF)
