# Supplementary figures and images for: The C-Terminal Sequence of RhoB Directs Protein Degradation through an Endo-Lysosomal Pathway
Source: PLoS One. 2009 Dec 2;4(12):e8117. doi: 10.1371/journal.pone.0008117 (PMC2780327; doi:10.1371/journal.pone.0008117)

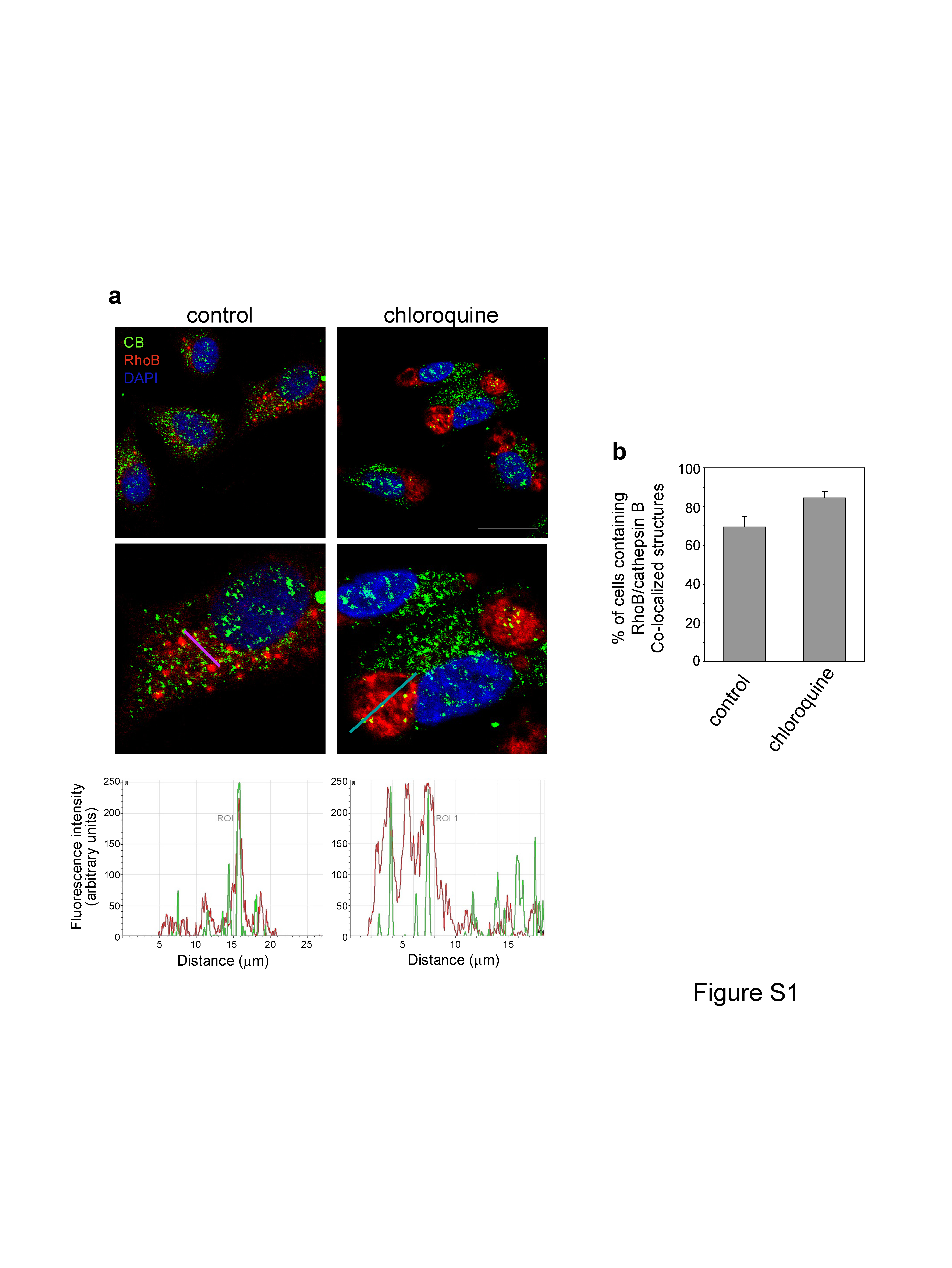

Supplement: Figure S1 — Co-localization of RhoB and lysosomal proteases. (a) The distribution of RhoB (red) and cathepsin B (CB, green) in BAEC treated with vehicle or 10 µM chloroquine for 24 h was assessed by IF, nuclei were stained with DAPI (blue); bar, 20 µm. Graphs represent the fluorescence intensity profile along the lines depicted in the images, showing the points of co-localization. Results are representative of three assays. (b) The percentage of cells showing five or more points of co-localization of RhoB and cathepsin B was calculated by monitoring 40 cells per experimental condition from three different experiments and it is shown as average values ± standard error of mean (SEM). (6.04 MB TIF) [file pone.0008117.s001.tif]

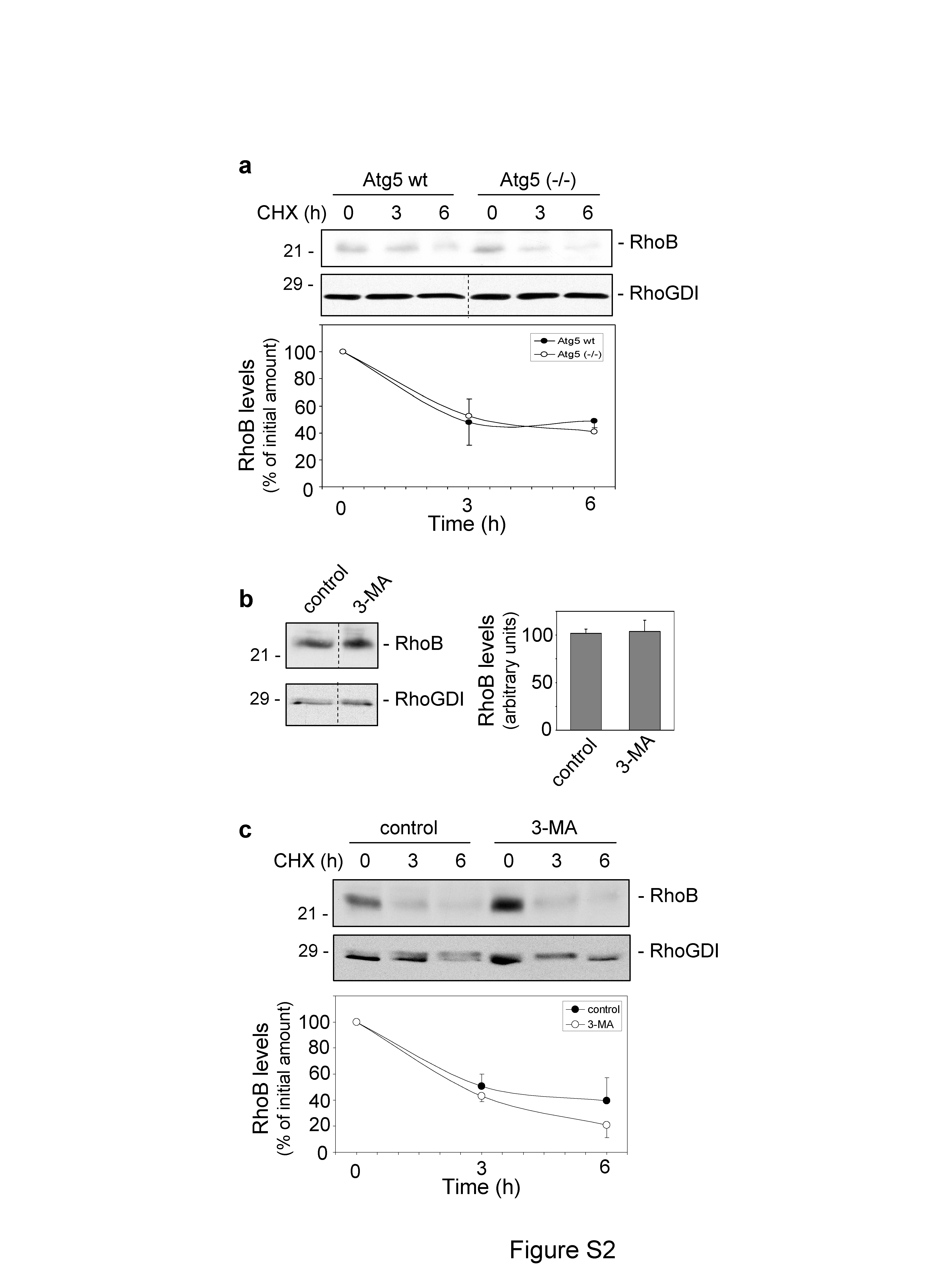

Supplement: Figure S2 — Effect of autophagy modulation on RhoB levels and degradation. (a) MEFs from wt and Atg5(−/−) mice were incubated with CHX to block new protein synthesis and the levels of endogenous RhoB were assessed at the indicated times by Western blot. (b) BAEC were treated in the absence or presence of 10 mM 3-methyladenine (3-MA) for 24 h and RhoB levels were estimated as above. Dotted lines show sites where lanes from the same gel have been cropped. Results are average values ± SEM of three experiments. (c) BAEC were pretreated with 3-MA for 30 min, after which, CHX was added and levels of RhoB were analyzed at the indicated times. Results are average values ± SEM of three experiments. (2.29 MB TIF) [file pone.0008117.s002.tif]

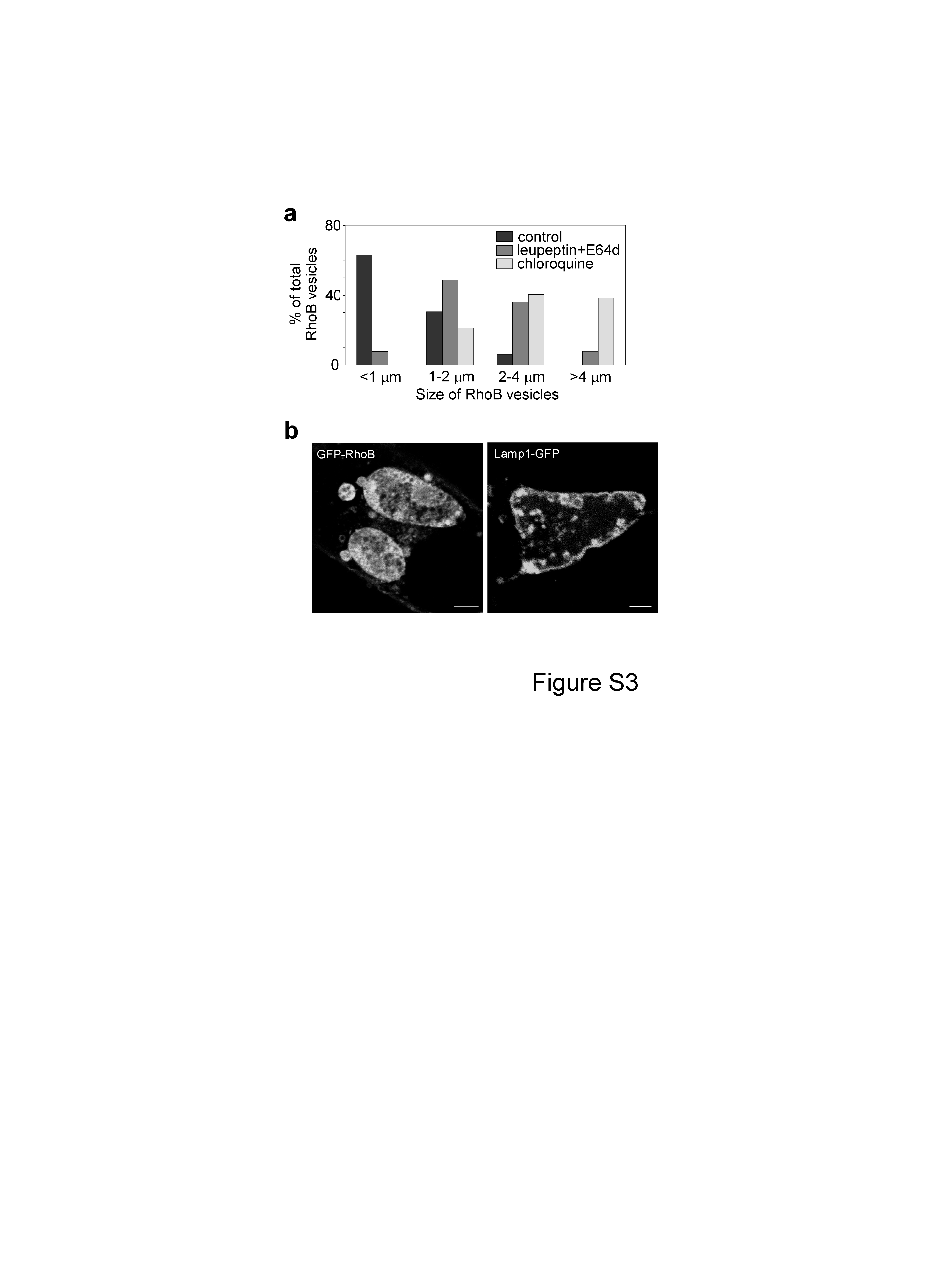

Supplement: Figure S3 — Effect of lysosomal inhibitors on the size and pattern of RhoB-positive vesicles. (a) BAEC were transfected with GFP-RhoB as in Fig. 2c and the size distribution of RhoB-positive vesicles was determined by measuring 60 randomly taken RhoB-positive vesicles per experimental condition from three experiments. The proportion of vesicles of the indicated sizes is expressed as percentage of the total. (b) Multivesicular pattern of chloroquine-elicited GFP-RhoB and Lamp1-GFP vesicles in BAEC transfected as in Fig. 2c. Bars, 5 µm. (1.92 MB TIF) [file pone.0008117.s003.tif]

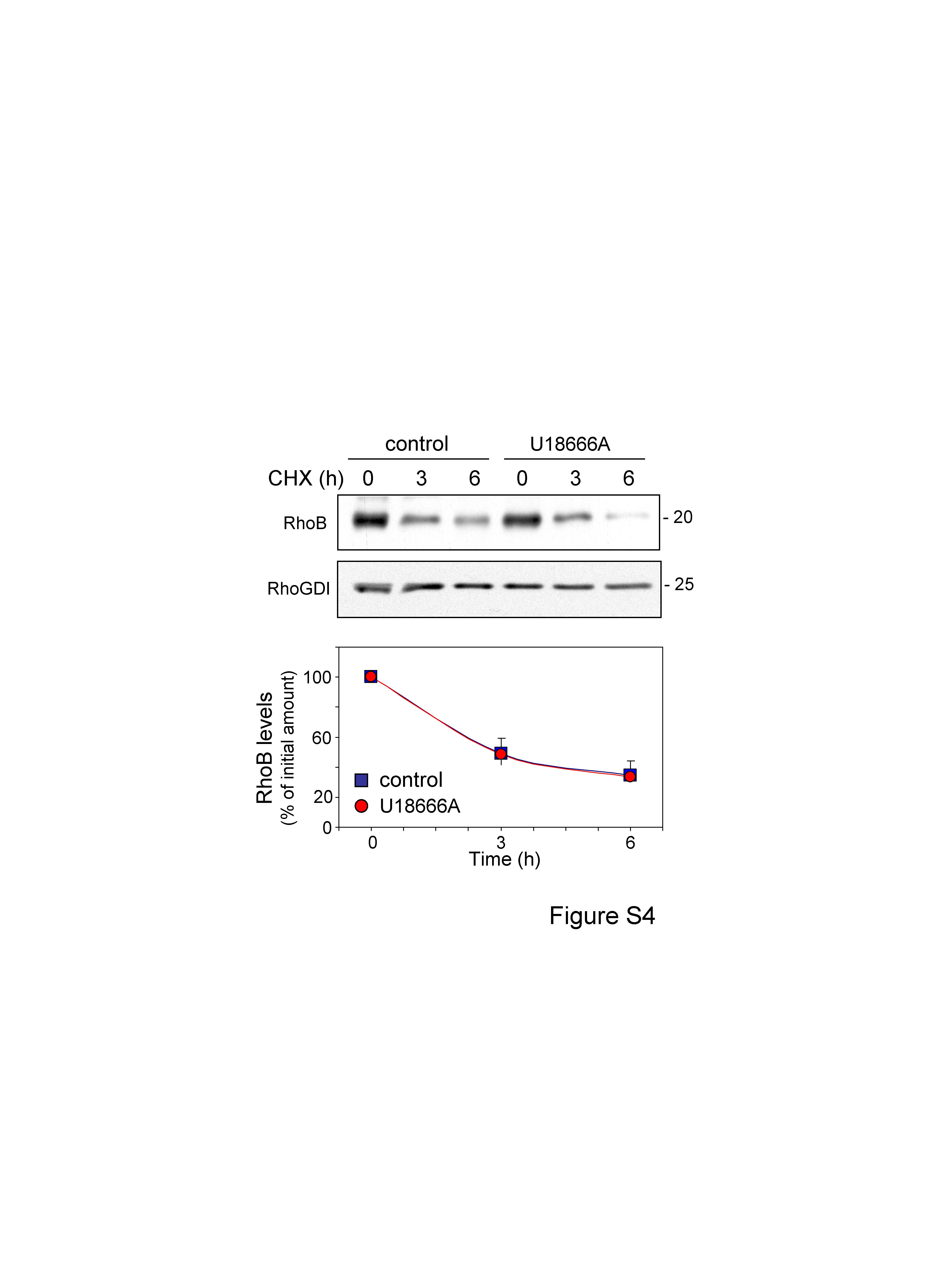

Supplement: Figure S4 — Effect of U18666A on RhoB stability. BAEC were treated with 10 µM U18666A for 24 h. Levels of endogenous RhoB and of RhoGDI (as control) were assessed by Western blot at the indicated times after CHX addition. Results shown are average values ± SEM of four experiments. (1.76 MB TIF) [file pone.0008117.s004.tif]

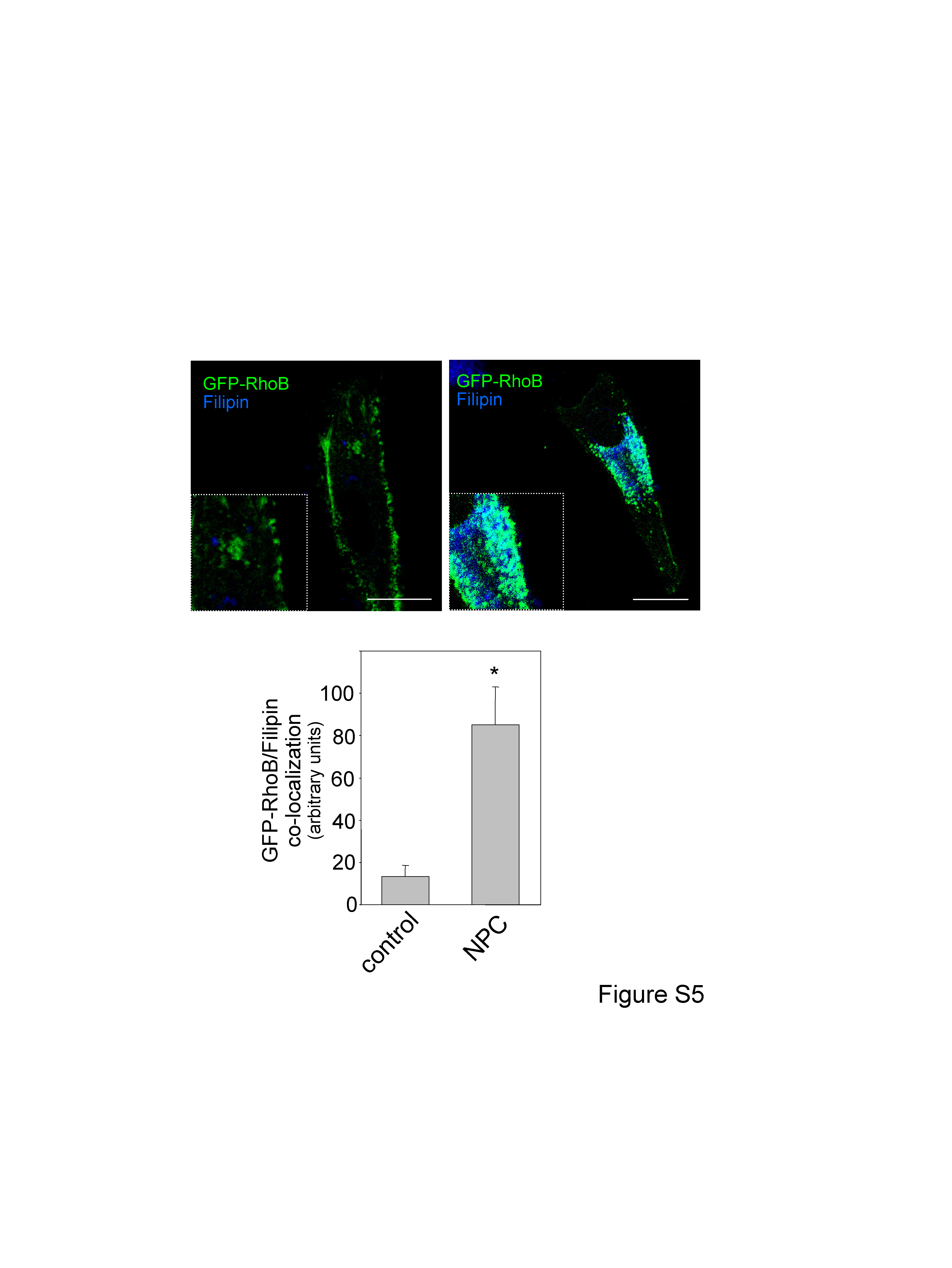

Supplement: Figure S5 — Co-localization of GFP-RhoB with cholesterol in control or NPC fibroblasts. Control or NPC fibroblasts were transfected with GFP-RhoB. Co-localization with cholesterol was evaluated in fixed cells stained with filipin. Bars, 20 µm. Co-localization is shown as average values ± SEM of determinations from three experiments (*p<0.05 vs control). (3.21 MB TIF) [file pone.0008117.s005.tif]

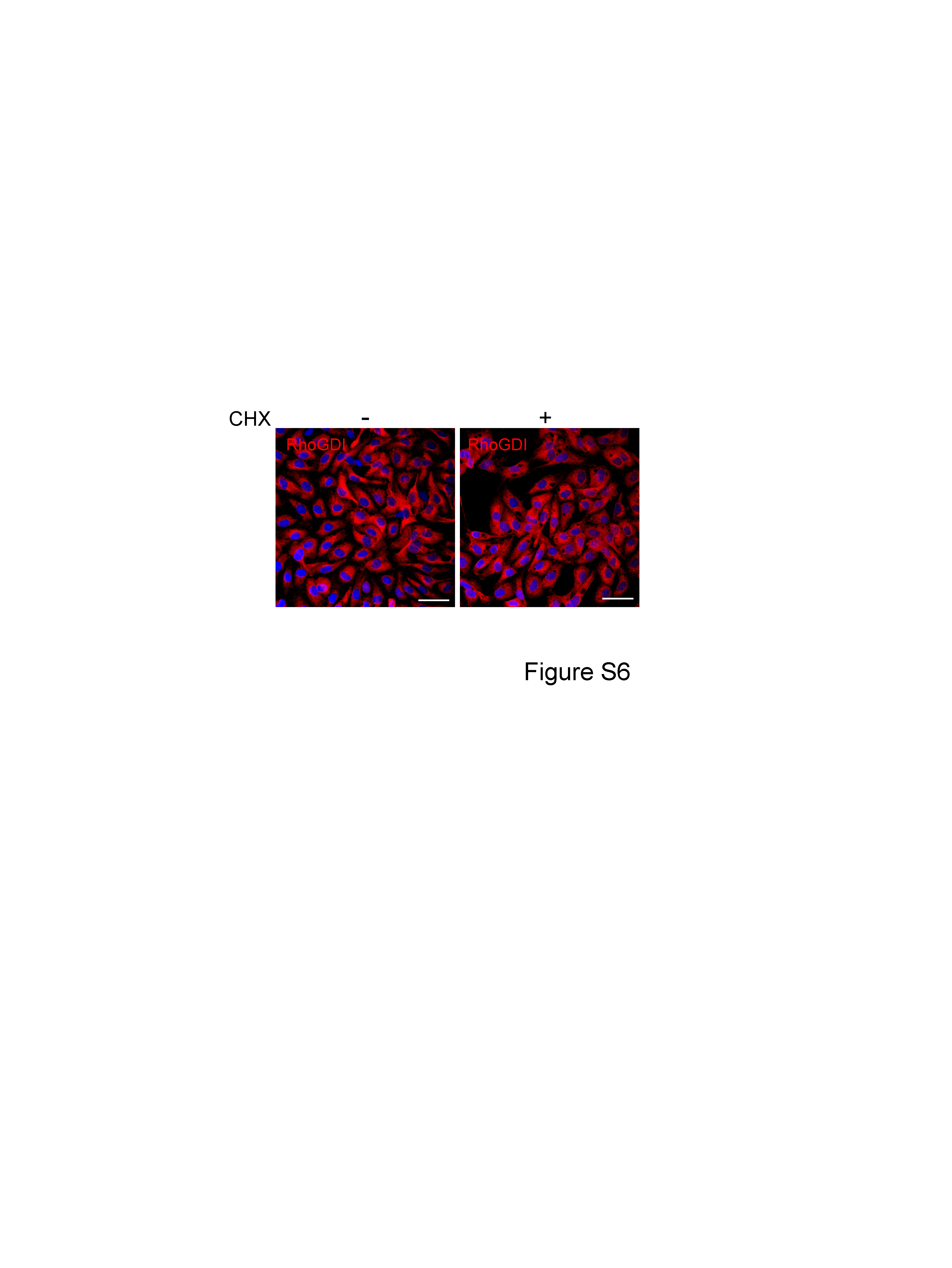

Supplement: Figure S6 — Levels of RhoGDI in BAEC detected by IF before and after CHX treatment. BAEC were treated as in Fig. 4, and fixed. Endogenous RhoGDI (in red) was detected by IF. Nuclei were stained with DAPI. Images shown are overall projections. Bars, 20 µm. (4.04 MB TIF) [file pone.0008117.s006.tif]

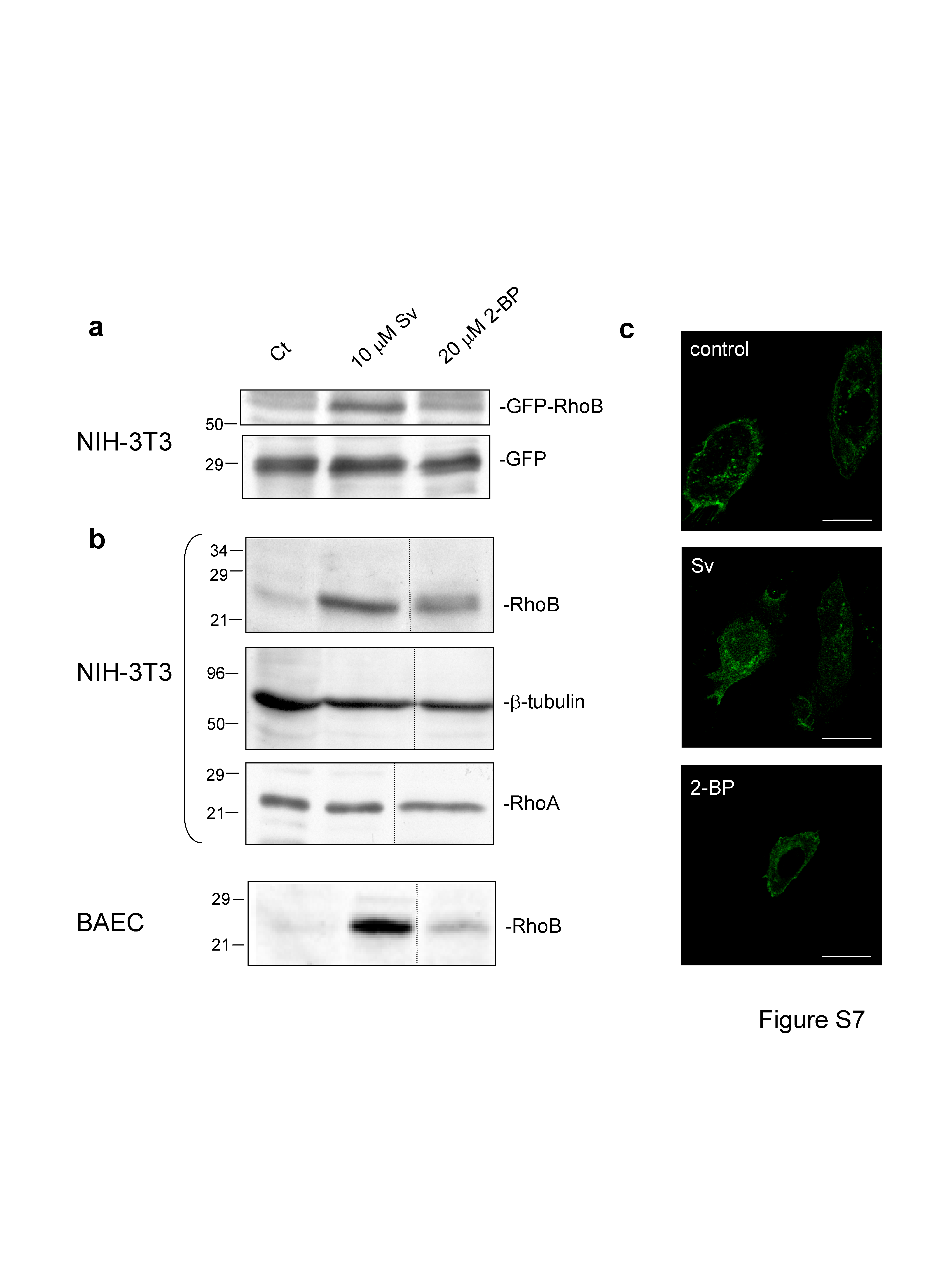

Supplement: Figure S7 — Effect of inhibitors of isoprenylation and palmitoylation on the levels of endogenous RhoB protein or of RhoB constructs in different cell types. (a) NIH-3T3 fibroblasts were transiently transfected with GFP or with GFP-RhoB, and construct levels were assessed by Western blot with an anti-GFP antibody after 24 h treatment with simvastatin (Sv) or 2-bromopalmitate (2-BP). (b) NIH-3T3 fibroblasts or BAEC were treated as in (a) and the levels of the indicated proteins were assessed by Western blot. Dotted lines show sites where lanes from the same gel have been cropped. (c) BAEC were transiently transfected with GFP-RhoB, treated with simvastatin or 2-BP for 3 h and visualized by live confocal microscopy. Note that in simvastatin-treated cells, GFP-RhoB can be detected in the nucleus, which is indicative of the presence of non isoprenylated construct. In contrast, in 2-BP-treated cells the appearance of GFP-RhoB is more diffuse than in control cells but the nucleus is excluded, as expected from an isoprenylated construct. Bars, 20 µm. (3.78 MB TIF) [file pone.0008117.s007.tif]

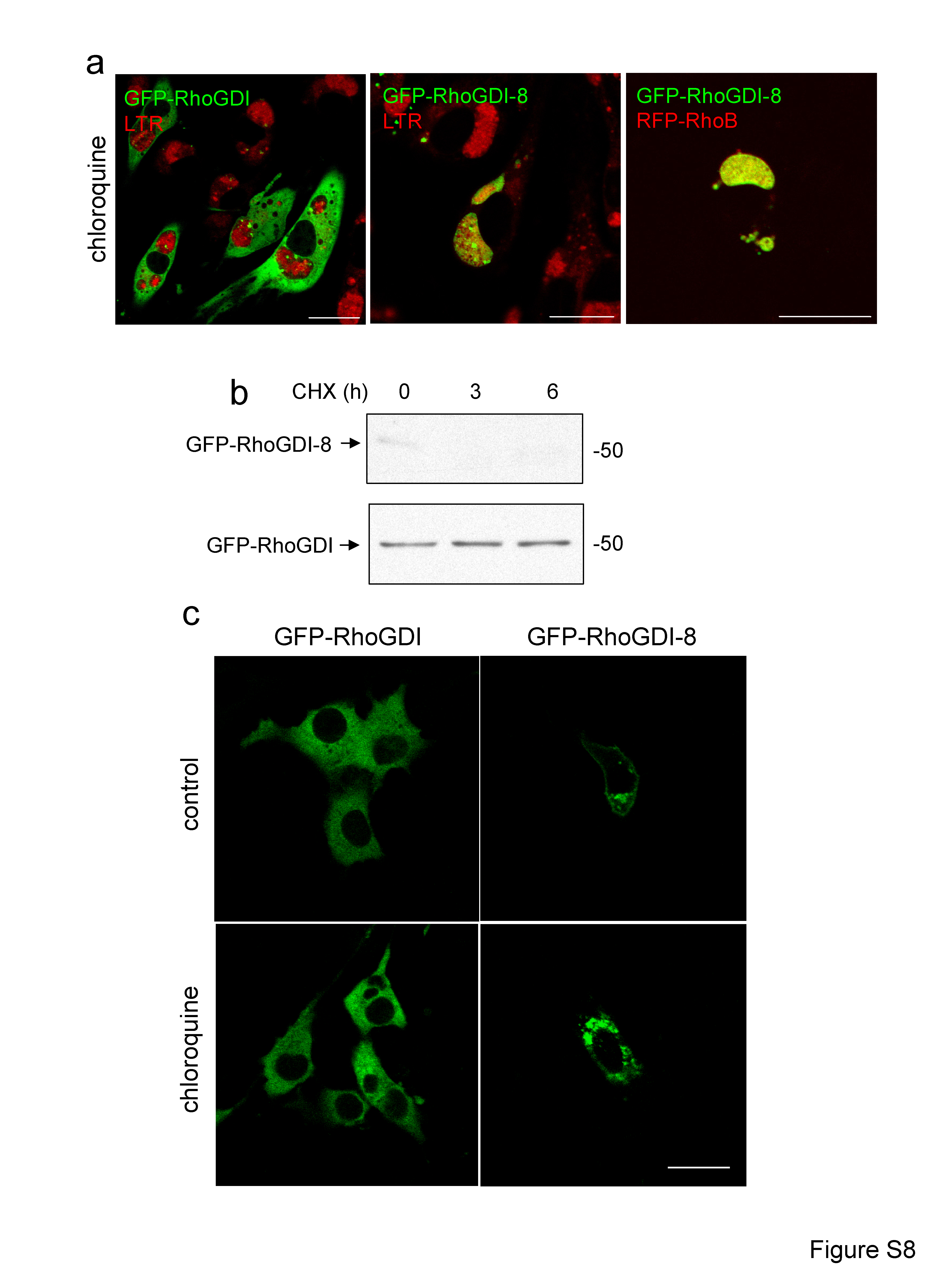

Supplement: Figure S8 — Cellular distribution and stability of GFP-RhoGDI and GFP-RhoGDI-8. (a) BAEC were transiently transfected with the indicated constructs as in Figure 6, treated with 10 µM chloroquine for 24 h and incubated with 25 nM LTR for 15 min before observation of live cells by confocal microscopy. Bar, 20 µm. (b,c) NIH-3T3 fibroblasts were stably transfected with the indicated constructs. Protein stability (b) and subcellular localization of constructs in control and chloroquine-treated cells (c) were assessed as described in Fig. 6, after 30 days in culture. Bar, 20 µm. (6.51 MB TIF) [file pone.0008117.s008.tif]

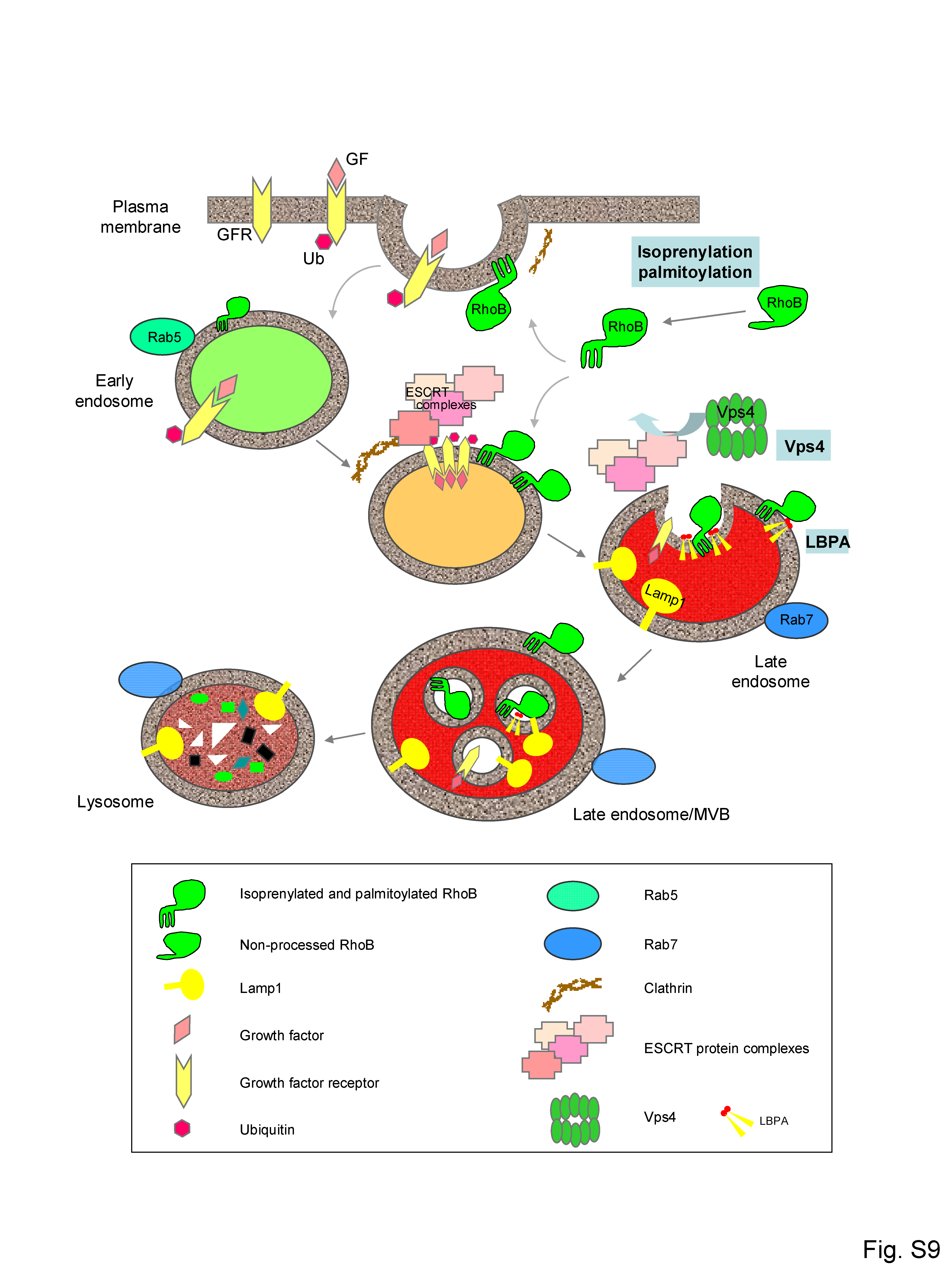

Supplement: Figure S9 — Hypothetical model for the degradation pathway of RhoB and -CINCCKVL chimeric proteins. Isoprenylated and palmitoylated RhoB is targeted to intracellular vesicles, mainly Rab7-, Lamp1-positive late endosomes, and to the plasma membrane. The association of the ESCRT complexes on the membrane of late endosomes promotes the invagination of the membrane. The ATPase Vps4 promotes the dissociation of the ESCRT proteins which is required for the intraluminal release of the invaginated vesicles. The late endosome-specific lipid LBPA promotes luminal vesicle formation due to its cone-shaped structure and controls endosome cholesterol content. Our results support a hypothesis according to which late-endosome associated RhoB may be delivered to intraluminal vesicles of MVB in a Vps4- and LBPA-dependent fashion, for degradation in acidic compartments. Inhibition of vesicular acidification with chloroquine or of lysosomal proteases with protease inhibitors results in the formation of dilated MVB in which RhoB accumulates. Genetic or pharmacological disruption of late endosome specific lipid dynamics also alters RhoB sorting. The proposed points of regulation of RhoB targeting to the endo-lysosomal pathway are shown in blue boxes. Based on our experimental observations, -CINCCKVL chimeric proteins would follow a similar pathway for rapid protein degradation. Ub, ubiquitin; GFR, growth factor receptor; GF, growth factor. (4.56 MB TIF) [file pone.0008117.s009.tif]
